# The Role of Alternating Bilateral Stimulation in Establishing Positive Cognition in EMDR Therapy: A Multi-Channel Near-Infrared Spectroscopy Study

**DOI:** 10.1371/journal.pone.0162735

**Published:** 2016-10-12

**Authors:** Tamaki Amano, Motomi Toichi

**Affiliations:** 1 Graduate School of Medicine, Kyoto University, Kyoto, Japan; 2 The Organization for Promoting Neurodevelopmental Disorder Research, Kyoto, Japan; UMR8194, FRANCE

## Abstract

Eye movement desensitisation and reprocessing (EMDR) is a standard method for treating post-traumatic stress disorder. EMDR treatment consists of desensitisation and resource development and installation (RDI) stages. Both protocols provide a positive alternating bilateral stimulation (BLS). The effect of desensitisation with BLS has been elucidated. However, a role for BLS in RDI remains unknown. Therefore, it is important to measure feelings as subjective data and physiological indicators as objective data to clarify the role of BLS in RDI. RDI was administered to 15 healthy volunteer subjects who experienced pleasant memories. Their oxygenated haemoglobin concentration ([oxy-Hb]), a sensitive index of brain activity, was measured from the prefrontal cortex (PFC) to the temporal cortex using multi-channel near-infrared spectroscopy during recall of a pleasant memory with or without BLS. The BLS used was alternating bilateral tactile stimulation with a vibration machine. The psychological evaluation suggested that RDI was successful. The results showed that, compared with non-BLS conditions, accessibility was increased and subjects were more relaxed under BLS conditions. A significant increase in [oxy-Hb] was detected in the right superior temporal sulcus (STS), and a decrease in the wide bilateral areas of the PFC was observed in response to BLS. The significant BLS-induced activation observed in the right STS, which is closely related to memory representation, suggests that BLS may help the recall of more representative pleasant memories. Furthermore, the significant reduction in the PFC, which is related to emotion regulation, suggests that BLS induces relaxation and comfortable feelings. These results indicate an important neural mechanism of RDI that emotional processing occurred rather than higher cognitive processing during this stage. Considering the neuroscientific evidence to date, BLS in RDI may enhance comfortable feelings about pleasant memories. Based on the current findings, the use of BLS in RDI may be warranted in some clinical situations.

## Introduction

It is common for people to experience severe stress immediately after a traumatic incident, but most people can resume their life within a few months. Most people are resilient and eventually recover from tragedy over time; however, some experience prolonged stress known as post-traumatic stress disorder (PTSD). PTSD is defined as a particular set of reactions that develop in people who have been through a traumatic event that threatened their life or safety and led to feelings of intense fear, helplessness, or horror. The main types of PTSD symptoms are re-experiencing the traumatic event, avoiding reminders of the trauma, and increased anxiety, emotional arousal, and negative cognitions, which occur automatically. There are some effective therapeutic methods, one of which includes the process of desensitising traumatic memories and establishing positive cognition. In the latter process, a client is usually asked to think about personally meaningful positive experiences that are associated with well-being. These personal positives may take the form of positive personal experiences of self-soothing, self-efficacy, self-acceptance, or courage. Thus, there are two cardinal components of the PTSD therapeutic process; one is to remove the traumatic memories, and the other is to establish positive cognition.

Eye movement desensitisation and reprocessing (EMDR) [1–2] is a standard method for treating PTSD and has been recommended by the American Psychiatric Association. This technique involves a unique procedure in which a therapist exposes the patient to bilateral stimulation (BLS), which involves alternating bilateral visual (eye movement), auditory, or sensory stimulation (e.g. tactile stimulation). The standard EMDR protocol consists of two main stages, desensitisation of traumatic memories and development and installation of a “resource”, such as safe and pleasant thoughts. The latter is called resource development and installation (RDI) [3–7]. In the standard protocol, both stages use alternating BLS. BLS is performed concurrently with the recall of the worst image of the trauma and the resources installation. RDI has become a powerful psychotherapeutic tool for relaxation and encompasses a wide range of resource development interventions during the stabilisation phase of PTSD treatment.

The EMDR protocol includes eight phases. Phase 1 involves gathering data about the patient’s history and clinical symptoms. Phase 2 establishes a therapeutic framework and defines the appropriate level of expectations. Phase 3 focuses on assessing trauma-related symptoms that involve vivid visual imagery associated with traumatic memories, positive and negative believe about self, relevant emotions, and bodily sensations. Phase 4 is the primary therapeutic stage, in which alternate BLS is used to ameliorate traumatic reactions. Phase 5 involves cognitive modification, in which positive cognition is installed. Phase 6 involves a scan of bodily sensations to identify pain or abnormal experiences. Phase 7 is the closure phase, and Phase 8 is used to prepare for the next session. RDI is typically used during the preparatory phases (Phases 2 and 3), but RDI can be attempted whenever the therapist feels it is appropriate.

Although the effect of BLS on desensitisation has been elucidated [8–11], the role of BLS in RDI [12] remains unknown. Hornsveld et al. reported that BLS decreases vividness, pleasantness, and experiences of strength of the intended quality or resource [13–14]. They suggested not using BLS in RDI because BLS may disturb RDI [15]. However, Leeds and Korn advocated the use of BLS as an important element in the standard RDI protocol [16]. Thus, whether BLS is beneficial for RDI is controversial. Our previous clinical studies showed that patients with chronic pain [17] (pain control protocol by Grant & Threlfo [18]) and those with dementia [19] were stabilised by BLS (alternating bilateral tactile stimulation on their hands or knees) during RDI treatment. We reported that BLS was a vital tool in RDI [17] [19]. In this study, to clarify the role of BLS in RDI, 15 subjects either received or did not receive BLS (i.e. alternating bilateral tactile stimulation with a machine) while recalling their own pleasant memories.

## Methods

### Ethics statement

These experimental procedures were approved by the local ethics committee of Kyoto University Graduate School of Medicine, and were carried out in accordance with the principles of the 1964 Declaration of Helsinki. The measures and procedures were explained to all subjects by the investigator, and all agreed and gave written informed consent before the start of the experiment.

### Subjects

Fifteen healthy volunteers (10 women and 5 men; mean age, 35.1 years, standard deviation: 11.0; range, 23–55 years) were included in the study. All were right-handed and had no other psychiatric complications, such as depression. Other demographic data of the subjects are presented in Table 1.

**Table 1:** Information pertaining to subjects.

N0.	Age	Gender	Occupation	Theme of resource
1	23	female	Student	happy home
2	24	female	Care giver	honor with work
3	26	male	Adviser	promise to marry
4	27	male	Office worker	first love
5	33	female	Care Manager	birth of my son
6	35	female	Dietitian	pass an examination
7	37	male	Care giver	pass an examination
8	39	female	Office worker	birth of my son
9	40	female	Care giver	prize winner
10	54	female	Care giver	wedding of daughter
11	55	female	Care Manager	grown-up daughter
12	25	male	Care giver	victory in volleyball game
13	24	male	Care giver	victory in baseball game
14	53	female	Care giver	wedding of son
15	22	female	Student	graduation ceremony
Ave.	34.5			
SD	11.3			

### Preparation for RDI

Each subject was interviewed to identify their “resource” 1 week before the experiment. The subjects were asked to think about personal and meaningful positive memories associated with a sense of well-being (e.g., pleasant or honourable memories). A short script was written for each individual, and it was read by an investigator during the near-infrared spectroscopy (NIRS) measurement so that the subject could easily recall the memory. The subjects’ “resource” themes are shown in Table 1.

### Procedures and Measurements of the effects of BLS

**Fig 1:**
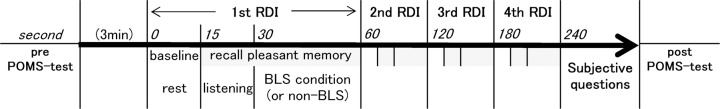
The design of the study. NIRS measurement procedure: Thick line indicates the NIRS measurement The POMS test was administered before and after the NIRS measurement.The baseline [oxy-Hb] level was the average value during the last 5 s of each 15-s rest period. Listened to the original script and recalled it for 15 s either accompanied by (BLS condition) or not accompanied by (non-BLS condition) BLS, two times each.Each subject was asked the subjective questions immediately after each of four RDI sessions.

The study procedure is shown in Fig 1. The Profile of Mood States (POMS) test [20] was administered before and after the experimental RDI session (n = 10).

The effects of BLS were examined in all subjects in two ways: by subjective questions and by measurement of cerebral activation using NIRS.

#### Subjective questions

Each subject was presented with the following prompt immediately after RDI sessions: “Which parts of BLS felt more effective, if you felt a difference?” They were provided with three choices (tactile stimulation/non-tactile stimulation/no difference) for use in responding to the following six items: (1) increased sensory detail, (2) increased accessibility, (3) increased vivid visual memory, (4) recall with emotion, (5) relaxation, and (6) experience of strength of “resource.” The mean results of the question were compared using Pearson's chi-square test. Differences with a *p*-value < 0.05 were considered significant, and a *p*-value <0.10 was considered a trend towards significance. The statistical analyses were performed with the SPSS software (ver. 22; SPSS, Inc., Chicago, IL, USA). The results of the questions are presented in Table 2.

**Table 2:** The results of the questions after RDI sessions. Upon completion of RDI sessions, subjects were immediately asked several questions. The results for the question “How did you feel with and without tactile stimulation?” are shown.

Question	tactile	non-tactile	no difference	*χ2*	*df*	*p*	
increased sensory detail	7	2	6	2.800	2	0.25	
increased accessibility	10	2	3	7.600	2	0.02	*
increased vividness	9	2	4	5.200	2	0.07	✝
recalled with emotion	8	0	7	0.067	1	0.80	
relaxed	12	0	3	5.400	1	0.02	*
strength of the resource quality	2	0	13	8.067	1	0.00	**

Chi-squared test (χ2 test)

** p<0.01

* p<0.05

✝p<0.1.

#### NIRS measurement

The RDI procedure was conducted as follows. The subject received the following instructions: “Listen to your original script and recall your pleasant memory, including sights, sounds, skin sensations, and smells.” After listening to the original script and imagining the memory for 15 s, a 30-s period of imagining randomly that either was or was not accompanied by BLS followed (i.e. one RDI session). BLS was conducted using a machine (Tac/Audio Scan; NeuroTek Corp., Wheat Ridge, CO, USA). A 15-s rest period between RDI sessions involved looking at a black point 2 m in front of the subject. Each subject underwent four RDI sessions under the two conditions (with and without BLS) while imagining the “resource” memory (Fig 1). The RDI sessions with or without BLS were presented in a random order.

### NIRS Data Processing and Analysis

Data were collected under two conditions: with BLS (BLS condition) and without BLS (non-BLS condition). Paired *t*-tests were used to examine differences in [oxy-Hb] changes between conditions in each brain region. A *p*-value <0.05 was considered significant. All analyses were performed with SPSS software (ver. 22; SPSS Inc., Chicago, IL, USA).

### NIRS Machine

**Fig 2:**
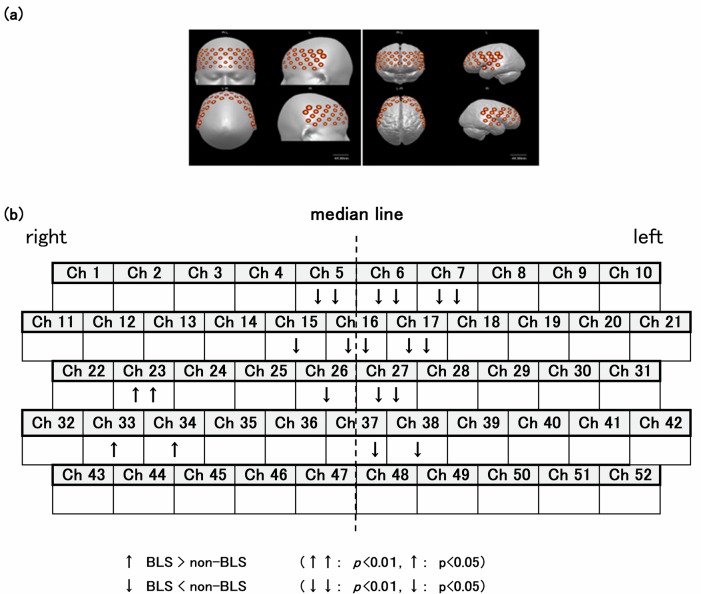
The probe position and the changes in [oxy-Hb]. (a) Position of the near-infrared spectroscopy (NIRS) probe and the area measured (Tsuzuki D et al., 2007). (b) Subtraction analyses of tactile stimulation minus non-tactile stimulation. The upward-pointing arrow, i.e. the symbol ↑, represents greater activation under the tactile stimulation than under the non-tactile stimulation, and the downward-pointing arrow, i.e. the symbol ↓, represents reduced activation under the tactile stimulation relative to the non-tactile stimulation. Repeated-measures *t*-tests were used to examine differences in the changes in [oxy-Hb] between conditions in each brain region. One upward-pointing arrow indicates a significance level of *p* < 0.05, and two indicate a significance level of *p* < 0.01 (i.e., ↑ *p* < 0.05, ↑↑ *p* < 0.01).

Cerebral activation was monitored by NIRS, which is an optical neuroimaging technology that allows for noninvasive detection of regional changes in blood flow [21–24]. A 52-channel ETG-4000 NIRS instrument (Hitachi Medical Corp., Tokyo, Japan) was used. The distance between the emitter and the detector was 30 mm. The mean depth measured was approximately 30 mm. Combinations of the 52 nearest-neighbour pairs of input and output fibres were used to obtain topographical images of areas from the frontal lobe to the temporal lobe. The measurement point was the centre point between the emitter and detector. The brain region corresponding to the probe is shown in Fig 2(A). The measurement points in the brain are shown in Fig 2(A). The centre of the lowest row of the 3 × 11 holder was placed on the Fpz (middle of the forehead), according to the International 10–20 system for electroencephalograms [25]. The probes in the lowest row were placed horizontally along the reference curve. The bottom edge of the probe was located on the top edge of the eyebrow [26]. Regional cerebral blood flow was measured as changes in oxygenated haemoglobin [oxy-Hb] and deoxygenated haemoglobin (deoxy-Hb). Changes in [oxy-Hb] are the most sensitive indicator of changes in regional cerebral blood flow and were used as the index of activation. Baseline [oxy-Hb] was set as the mean value during the last 5 s of each rest period just before the RDI procedure. The changes in [oxy-Hb] were measured every 0.1 s. All measurements were recorded using a video camera.

### The POMS test

The POMS test is a standardised validated psychological test formulated by McNair et al. [20] that measures how subjects feel before and after RDI. Mean POMS scores (n = 10) before and after RDI were compared using paired *t*-tests.

## Results

All relevant data are within the paper and its Supporting Information files.

### The POMS test

**Fig 3:**
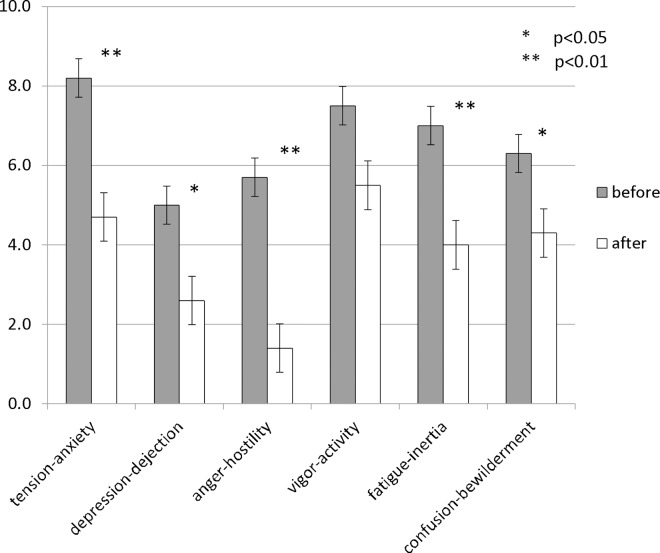
Changes in POMS scores obtained after compared with those obtained before the session (n = 10). The paired *t*-test was used to compare test scores before and after the session (p < 0.05: *, p < 0.01: **).

The results are presented in Fig 3. The mean POMS scores after RDI were lower than those before RDI (tension-anxiety: 8.2 and 4.7, respectively; depression-dejection: 5.0 and 2.6, respectively; anger-hostility: 5.7 and 1.4, respectively; fatigue-inertia: 7.0 and 4.0, respectively; confusion-bewilderment: 6.3 and 4.3, respectively; and vigour-activity: 7.5 and 5.5, respectively). The paired *t*-test results revealed significant differences (*p* < 0.05) for the tension-anxiety (*t* = 4.20, *p* = 0.02), depression-dejection (*t* = 2.27, *p* = 0.049), anger-hostility (*t* = 2.99, *p* = 0.015), fatigue-inertia (*t* = 3.30, *p* = 0.009), and confusion-bewilderment (*t* = 2.53, *p* = 0.032) scores. These changes indicated that RDI resulted in a better subjective mood, reflecting an increase in positive feelings.

### The effect of BLS on answers to subjective questions

All 15 subjects completed RDI (Table 2). BLS increased “accessibility” [*χ*^*2*^ (2, n = 15) = 7.60, *p* = 0.022] and “relaxation” [*χ*^*2*^ (1, n = 15) = 5.40, *p* = 0.020] significantly. “Increased vividness” [*χ*^*2*^ (2, n = 15) = 5.20, *p* = 0.074] showed a trend towards significance. Regarding the “strength of resource quality,” the number of the answers stating “no difference” was significantly greater than that of “yes” or “no” [*χ*^*2*^ (1, n = 15) = 8.067, *p* = 0.005], meaning there was no significant difference between BLS and non-BLS conditions. Therefore, subjects did not feel any difference between the BLS and non-BLS conditions.

### NIRS data: [oxy-Hb] subtraction analysis (BLS − non-BLS)

There was more activation under the BLS condition than under the non-BLS condition in Ch 23 (*t* = 3.33, *p* = 0.005; Brodmann’s area, BA22), Ch 33 (*t* = 2.16, *p* = 0.048; BA22), and Ch 34 (*t* = 2.70, *p* = 0.017; BA21); Ch 23/33/34 collectively correspond to the right superior temporal sulcus (STS) (BA21/22). However, there was less activation under the BLS condition in Ch 5 (*t* = −3.46, *p* = 0.004; BA9), Ch 6 (*t* = −4.98, *p* = 0.000; BA8/9/10), Ch 7 (*t* = −3.92, *p* = 0.002; BA9/46), Ch 15 (*t* = −2.50, *p* = 0.026; BA8–10), Ch 16 (*t* = −4.13, *p* = 0.001; BA9), Ch 17 (*t* = 4.24, *p* = 0.001; BA8–10), Ch 26 (*t* = −2.95, *p* = 0.011; BA8/9), Ch 27 (*t* = −3.01, *p* = 0.009; BA8–10), Ch 37 (*t* = −2.90, *p* = 0.012; BA10), Ch 38 (*t* = −2.45, *p* = 0.028; BA8–10). Ch 5/6/15/16/17/26/27 together correspond to the bilateral medial prefrontal cortex (MPFC) (BA8/9/10) and Ch 7 corresponds to the left dorsolateral prefrontal cortex (DLPFC) (BA9/46); Ch 37/38 correspond to the left orbitofrontal cortex (OFC) [26].

## Discussion

According to the POMS data, the RDI experimental sessions successfully increased subjects’ positive feelings. The effect of BLS was examined using both the subjective questions and the NIRS data.

The results of the subjective questions revealed that BLS facilitated easier access to positive memories, leading to a significantly more relaxed state. Increase in vividness showed a trend towards significance between BLS and non-BLS (Table 2).

According to the NIRS data, the [oxy-Hb] level showed greater activation in wide areas of the bilateral temporal regions under the BLS compared with the non-BLS condition. The BLS may symmetrically activate the secondary sensory cortex in the bilateral temporal lobes [27, 28]. A significant increase in [oxy-Hg] in the right STS and a significant decrease in [oxy-Hg] in the bilateral MPFC, the left OFC, and the left DLPFC were observed only under the BLS condition (Table 3, Fig 3(B)). We will discuss the possible effects of RDI based on the four above-mentioned cerebral regions that showed significant changes.

**Table 3:** Changes in [oxy-Hb] (BLS vs. non-BLS)

Channel No.	average	Standard deviation	t	df	p
5	-.043	.048	-3.46	14	.004**
6	-.046	.036	-4.98	14	.000**
7	-.047	.046	-3.92	14	.002**
15	-.034	.053	-2.50	14	.026*
16	-.050	.047	-4.13	14	.001**
17	-.062	.056	-4.24	14	.001**
23	.096	.112	3.33	14	.005**
26	-.045	.059	-2.95	14	.011*
27	-.051	.066	-3.01	14	.009**
33	.071	.127	2.16	14	.048*
34	.078	.112	2.70	14	.017*
37	-.101	.135	-2.90	14	.012*
38	-.073	.115	-2.45	14	.028*

Paired t-test

** p<0.01

* p<0.05

The first finding was a significant increase in [oxy-Hb] in the right STS during BLS. The role of the STS in human cognitive functioning has been elucidated [29]. Recent studies have reported a relationship between the STS and memory representation [30], as first described in Penfield’s classic work [31], whereas the right STS is more highly activated than the left during visual recall [32] [33]. In this study, the right STS was significantly activated by BLS (using alternating bilateral tactile stimulation) during recall of pleasant memories. Interestingly, our previous study of unpleasant memories showed significantly *reduced* activity in the right STS during recall with BLS (using eye movement) [32] [33] [in submission]. The seemingly contradictory findings regarding the response of the STS may be attributable to differences in the nature of pleasant and unpleasant memories, considering that the right STS is associated with memory representation. The increase in [oxy-Hb] due to BLS may reflect the reported “increased accessibility” and “increased vividness” in the subjective questions.

The next finding was decreased activity in the MPFC, OFC, and DLPFC with BLS. A significant decrease in [oxy-Hb] was revealed in the bilateral MPFC during BLS. The role of MPFC has been suggested to be inhibition of amygdala activation [34] or self-reflection (how one thinks about oneself), which is a defining feature of the human mind [35]. Self-reflection is thought to draw on a shared cognitive process that may not be based on real memories. The reduced activity in response to BLS suggests that the ability to inhibit the amygdala and engage in self-reflection is reduced BLS compared with non-BLS. Therefore, it may be possible to recall an actual pleasant memory more easily and with greater emotion. A significant decrease in [oxy-Hb] was also revealed in the medial and left OFC. The OFC is important in unpleasant emotional responses and arousal [36] [37] [38] as well as emotional regulation [39]. According to our previous studies [32, 33, in submission], a high level of [oxy-Hb] in the OFC area is correlated with the occurrence of an unpleasant emotion. Therefore, the increased blood flow in the OFC is considered to reflect a negative emotional state attributable to an unpleasant memory. In this study, a reduced [oxy-Hb] level in response to BLS reflected reduced negative emotions during BLS. Lastly, the [oxy-Hb] level in the left DLPFC also decreased significantly in response to BLS. The DLPFC plays a role in generating flexible and adaptive behaviour in accordance with current sensory input [40, 41]. In our previous case studies [32, 33], no significant changes were observed during EMDR sessions in patients with PTSD using BLS. However, in this study, a significant decrease in [oxy-Hb] was revealed in the left DLPFC. These results suggest that subjects do not need to use the left DLPFC for regulation or thinking during BLS. Therefore, they may be more relaxed by the BLS compared with those in the non-BLS condition, as shown by the answers to the subjective questions. Because humans experience and understand the world through signals received by the senses and interpreted by the brain [42].

## Conclusions

Clear differences between using and not using BLS were observed based on the subjective interview and the NIRS biological data. Our results indicated that using BLS increased the effectiveness of RDI over not using BLS.

## Limitations

This study had several limitations. First, we used NIRS, which does not measure deep brain areas (e.g., limbic areas, such as the amygdala), and only measures a narrow region from the PFC to the inferior temporal lobe. Thus, the data we obtained may not precisely match those obtained via positron emission tomography, single-photon emission computed tomography, or functional magnetic resonance imaging. NIRS may be useful as an objective monitoring tool in clinical situations. Second, our sample was small, and more subjects need to be examined to confirm the present findings and clarify the relevant mechanisms of action. A comparison between BLS using tactile stimulation and that using eye movement is needed in a future study.

## Supporting Information

S1 Table The answers to the subjective questions in each subject. (TIF)

S2 Table Changes of [oxy-Hb] in each subject. (TIF)

S3 Table The POMS data in each subject. (TIF)
