# Supplementary figures and images for: The Role of Alternating Bilateral Stimulation in Establishing Positive Cognition in EMDR Therapy: A Multi-Channel Near-Infrared Spectroscopy Study
Source: PLoS One. 2016 Oct 12;11(10):e0162735. doi: 10.1371/journal.pone.0162735 (PMC5061320; doi:10.1371/journal.pone.0162735)

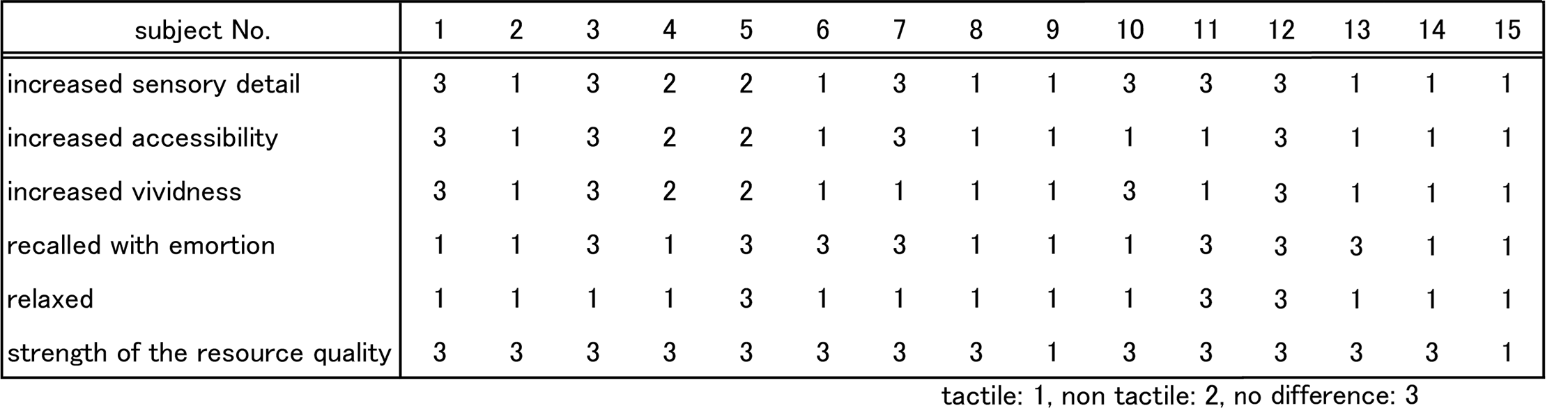

Supplement: S1 Table — (TIF) [file pone.0162735.s001.tif]

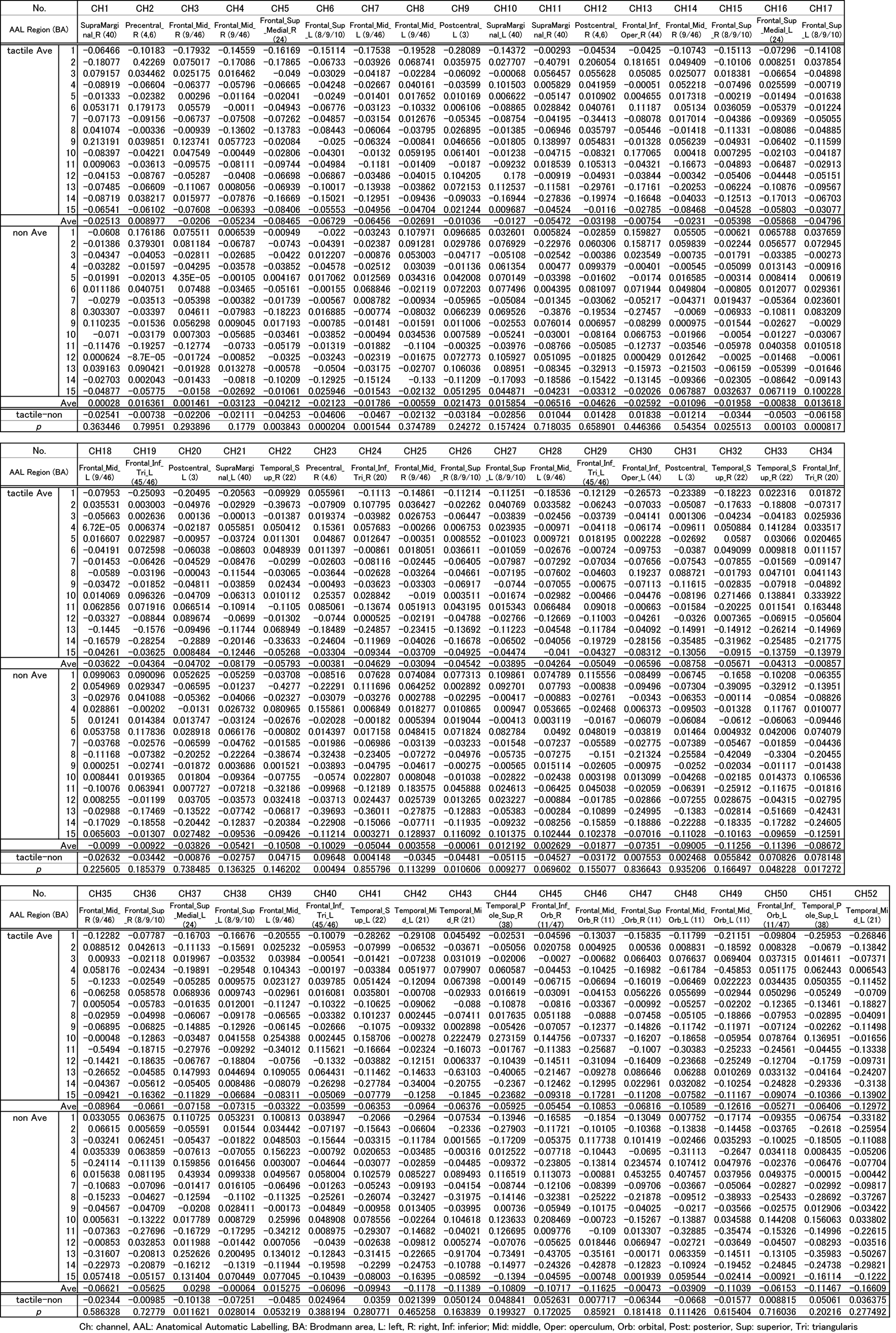

Supplement: S2 Table — (TIF) [file pone.0162735.s002.tif]

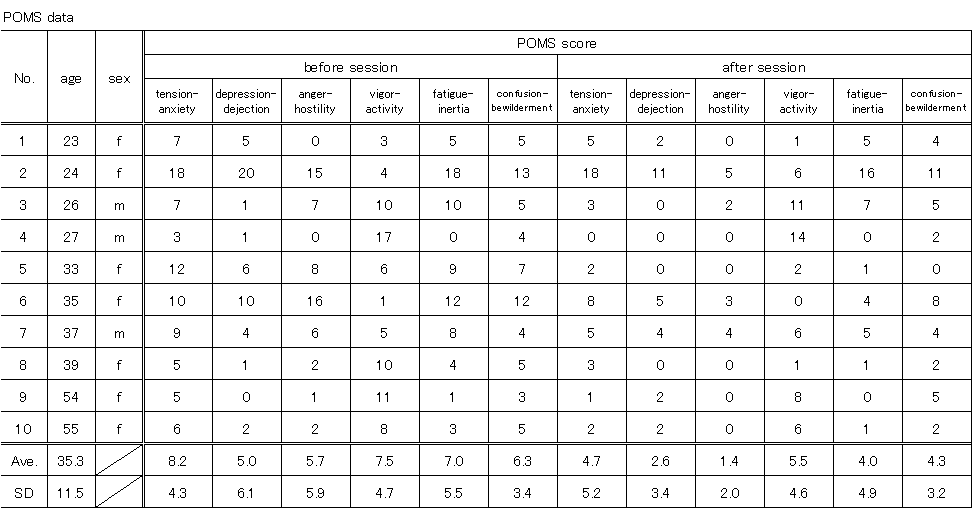

Supplement: S3 Table — (TIF) [file pone.0162735.s003.tif]
